# Extended Hückel Semi-Empirical Approach as an Efficient Method for Structural Defects Analysis in 4H-SiC

**DOI:** 10.3390/ma14051247

**Published:** 2021-03-06

**Authors:** Janusz Wozny, Andrii Kovalchuk, Jacek Podgorski, Zbigniew Lisik

**Affiliations:** 1 Department of Semiconductor and Optoelectronic Devices, Lodz University of Technology, Wolczanska 211/215, 90-924 Lodz, Poland; andrii.kovalchuk@dokt.p.lodz.pl (A.K.); jacek.podgorski@p.lodz.pl (J.P.); zbigniew.lisik@p.lodz.pl (Z.L.); 2 Optical Fiber and Cable Technology, Corning Optical Communications Polska, Smolice 1e, 95-010 Strykow, Poland

**Keywords:** 4H-SiC, defects, simulation, atomic structure, extended Hückel, semi-empirical

## Abstract

This paper presents an efficient method to calculate the influence of structural defects on the energy levels and energy band-gap for the 4H-SiC semiconductor. The semi-empirical extended Hückel method was applied to both ideal 4H-SiC crystal and different structures with defects like vacancies, stacking faults, and threading edge dislocations. The Synopsys QuatumATK package was used to perform the simulations. The results are in good agreement with standard density functional theory (DFT) methods and the computing time is much lower. This means that a structure with ca. 1000 atoms could be easily modeled on typical computing servers within a few hours of computing time, enabling fast and accurate simulation of non-ideal atomic structures.

## 1. Introduction

Silicon carbide (SiC) and its biggest competitor, gallium nitride (GaN), are the wide band-gap semiconductors that are the workhorses of the modern power electronic industry. Due to their large energy band-gap, power devices composed of these materials can sustain a much higher critical electric field and a higher temperature than similar silicon structures. GaN is mostly used for GaN-on-something geometries, like GaN-on-Si and GaN-on-sapphire [1] as there are no commercially available GaN substrates. Thus, from this point of view, SiC technology seems to be more mature. Currently available SiC substrates are 6” diameter wafers [2]. Most of them are 4H-SiC polytype substrates, since the 4H-SiC hexagonal atomic structure offers the highest energy band-gap and thermal conductivity. For a long time, SiC technology suffered from the presence of micropipes, which is a macroscopic structural defect [3]. Nowadays, this problem has been eliminated, but additional structural defects may deteriorate the parameters of manufactured devices. These other defects include carbon vacancies $V_{C}$ [4], stacking faults [5], and other structural defects such as threading edge dislocations (TEDs), screw dislocations (SDs), and basal plane dislocations (BPDs). Among these defects, some defects can be revealed just after substrate fabrication (TEDs, SDs, and BPLs), but there are also killing defects, like Shockley stacking faults (SSFs), which can occur during the operation of the device, causing malfunction of the electronic unit. To investigate the physics of the defects, an approach should be used that considers the atomic structure and that is computationally efficient. To perform atomic-scale simulations, different types of the ab initio density functional theory (DFT) approach are used. There are many commercial (MedeA [6], VASP [7], and QuatumATK [8]) and open-source tools (e.g., QuantumExpresso [9]) that use this method. The main problem is that with this method, due to simulation time and memory requirements, it is challenging to analyze structures with more than 200–300 atoms. This is not a problem for point defects, but for larger structural defects, e.g., TEDs, the modeling cannot be effectively performed. We wanted to show that it is possible to deal with this problem if the semi-empirical method is used. The semi-empirical approach is derived from DFT [10]. We wanted to verify if the approximations applied would influence the simulation results. This paper focuses on calculating the density of states (DOS) for ideal and defected 4H-SiC. The simulations were performed using Synopsys QuatumATK [8] with the semi-empirical approach based on the extended Hückel method with a set of parameters appropriate for 4H-SiC.

## 2. Simulation Software and Models

Since an efficient approach for DOS calculation is required, we focused on semi-empirical methods. A detailed analysis of the computational efficiency of different approaches is presented in [8]. QuatumATK offers two semi-empirical tight-binding methods for calculating the Hamiltonian of the system: the Slater–Koster model [11] and the extended Hückel model [12]. The semi-empirical extended Hückel (SEeH) is a semi-empirical approach; thus, it may not always receive the same respect from computational physics researchers as pure ab initio models. However, the electronics industry requires an efficient yet accurate approach to investigate atom-scale structures. Thus, semi-empirical models are still being investigated. The SEeH model was successfully used to simulate the elementary cells of several SiC polytypes [13]. The SEeH approach was also used by de Souza et al. [14] to investigate group-IV element nanosheets. In that work, the biggest concern was also the reliability of the semi-empirical model. The general conclusion was that the extended Hückel agrees very well with DFT calculations, including structures with point defects. The applicability and the theory behind the extended Hückel model was reviewed [15], where this approach was summarized as a method for “efficient computations on large-scale systems”.

QuatumATK offers two semi-empirical tight-binding methods for calculating the Hamiltonian of the system: the Slater–Koster model [11] and the extended Hückel model [12]. The SEeH model implemented in QuantumATK appears to offer higher numerical stability for 4H-SiC calculation than the Slater–Koster approach. The QuantumATK default Cerda parameters [16] designated for SiC were assumed. The DOS values presented in this article were calculated as the total projected density of states (PDOS). Two methods can be used to calculate PDOS: the tetrahedron method [17] and Gaussian smearing [18]. Both of them were used depending on the size of the atomic structure.

## 3. Ideal 4H-SiC

### 3.1. Energy Band-Gap

To verify the accuracy of the SEeH model, we calculated the band structures for the elementary cells of 3C-, 4H-, and 6H-SiC bulk crystals. These are the polytypes that have been under investigation by the electronic semiconductor industry for many years. The lattice constants of elementary cells are as follows: 3C: a=b=c=4.3485 Å; 4H: a=b=3.073 Å and c=10.053 Å; 6H: a=b=3.073 Å and c=15.117 Å. We decided to keep the same cells’ dimensions fixed across all methods considered. We verified that regardless of the relaxation method used, the hybrid-functional calculations with plane-wave basis sets (PW-HSE), the linear combination of atomic orbitals with Perdew- Burke- Ernzerhof functionals for solids (LCAO-PBEs), or Force Field, the band-gap for the relaxed cell did not differ more than 2% with respect to the fixed dimensions elementary cell. The values of the energy band-gap $E_{g}$ are listed in Table 1. As reference data, we quoted values of $E_{g}$, which can be found in the literature [19]. Besides the semi-empirical model, we calculated the band structures and $E_{g}$ using two DFT models: (i) the hybrid-functional calculations with plane-wave basis sets (PW-HSE) [20] and (ii) the hybrid-functional method for linear combination of atomic orbitals (LCAO-HSE) [20]. PW-HSE provides the most accurate and reliable values of $E_{g}$ [8]; however, PW-HSE is the most computationally demanding approach. LCAO-HSE is claimed to have similar accuracy as PW-HSE at a much lower computational cost [20].

**Figure 1:**
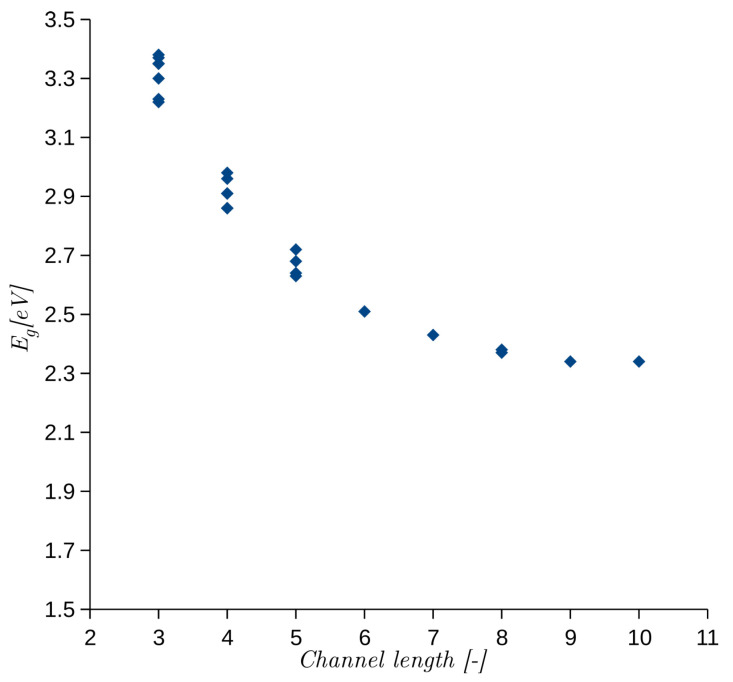
$E_{g}$ obtained from semi-empirical extended Hückel (SEeH) calculations as a function of interstitial channel length. The length is defined as a number of atomic bilayers [22].

In a series of publications [21,22,23], the authors investigated and explained the cause of different $E_{g}$ for SiC polytypes. They linked $E_{g}$ with the length of an interstitial channel, which may be distinguished in the crystal structures of different polytypes. To verify the SEeH model, we calculated the $E_{g}$ for the same 24 stacking sequences given in [23]. The calculated $E_{g}$ as a function of interstitial channel length is shown in Figure 1. The dependence is exactly the same as reported in [23].

### 3.2. Density of States Function

**Figure 2:**
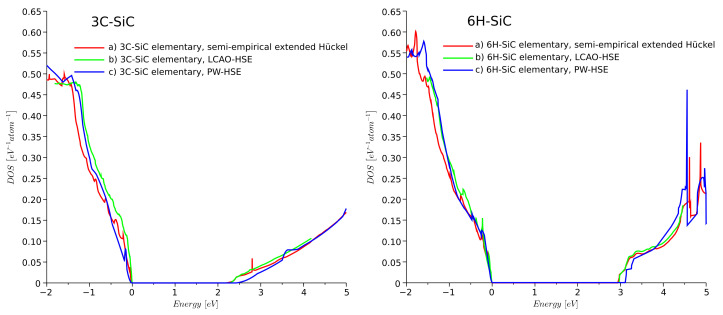
Density of states (DOS) of ideal 3C- and 6H-SiC obtained from QuantumATK using different models: (a) semi-empirical extended Hückel, (b) LCAO-HSE, and (c) PW-HSE models.

To verify the semi-empirical model, the DOS was calculated for the elementary cells of ideal 3C-, 4H-, and 6H-SiC crystal. The DOS for 3C- and 6H-SiC was calculated using (a) the semi-empirical extended Hückel, (b) the LCAO-HSE, and (c) the PW-HSE models. The shape, magnitude, and $E_{g}$ were found to be similar for all three approaches, as shown in Figure 2. The computational time for SEeH was at least three orders of magnitude lower than for PW-HSE.

Since we wanted to focus on 4H-SiC, we compared more models for this crystal. Additionally, we considered results obtained from MedeA-VASP [6] using the DFT framework with the meta-generalized gradient approximation (Meta-GGA) with the modified Becke Johnson functional combined with local density approximation (MBJLDA) approach [24,25].

**Figure 3:**
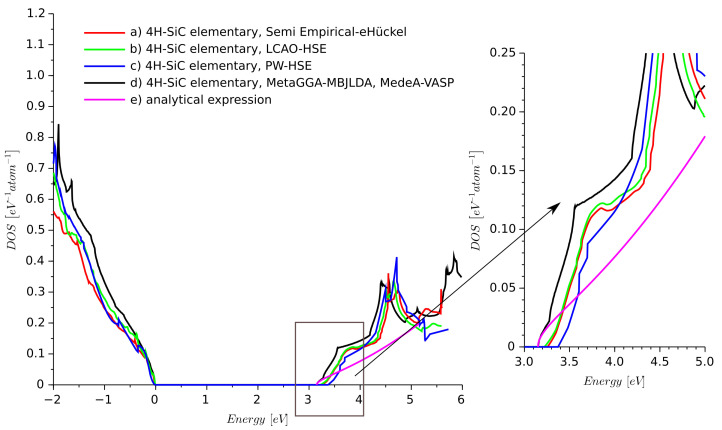
Density of states of ideal 4H-SiC obtained from different methods: (a) semi-empirical extended Hückel, (b) LCAO-HSE, (c) PW-HSE, (d) meta-generalized gradient approximation using modified Becke Johnson functional combined with local density approximation (MetaGGA-MBJLDA), MedeA-VASP software, and (e) analytical expression for electron DOS.

We used MBJLDA here since it provides an accurate value of the energy band-gap of semiconductors compared to other LDA/GGA methods [25]. The calculated DOS is shown in Figure 3. The results obtained from the extended Hückel semi-empirical method are very similar. The presented comparison shows that the semi-empirical model does not provide a DOS for elementary 4H-SiC much different than provided by the different types of DFT.

**Figure 4:**
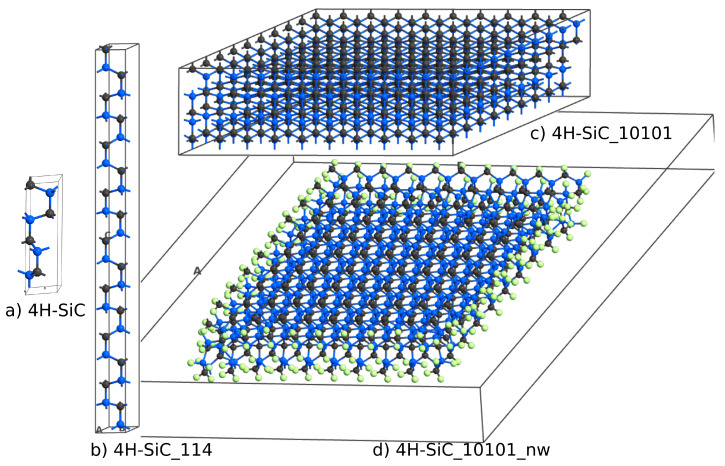
Atomic structures of ideal 4H-SiC (a) elementary cell, (b) 1×1×4 supercell, (c) 10×10×1 supercell, and (d) a 10×10×1 4H-SiC nanowire with side walls passivated by hydrogen atoms.

The next question was if a similar DOS could be obtained for large 4H-SiC supercells. To answer this question, we considered three different supercells, as shown in Figure 4. The elementary cell of 4H-SiC, shown in Figure 4 and labeled 4H-SiC, consists of eight atoms. 4H-SiC_114 is a supercell built from 1×1×4 elementary cells, 4H-SiC_10101 is a 10×10×1 supercell, and 4H-SiC_10101_nw is a 10×10×1 nanowire with edges passivated by hydrogen atoms. To create a nanowire structure, a 10 Å gap was introduced between the periodic boundary condition and the sidewall of the SiC domain. The two most external Si–C side layers together with hydrogen atoms were relaxed using the force-field approach.

**Figure 5:**
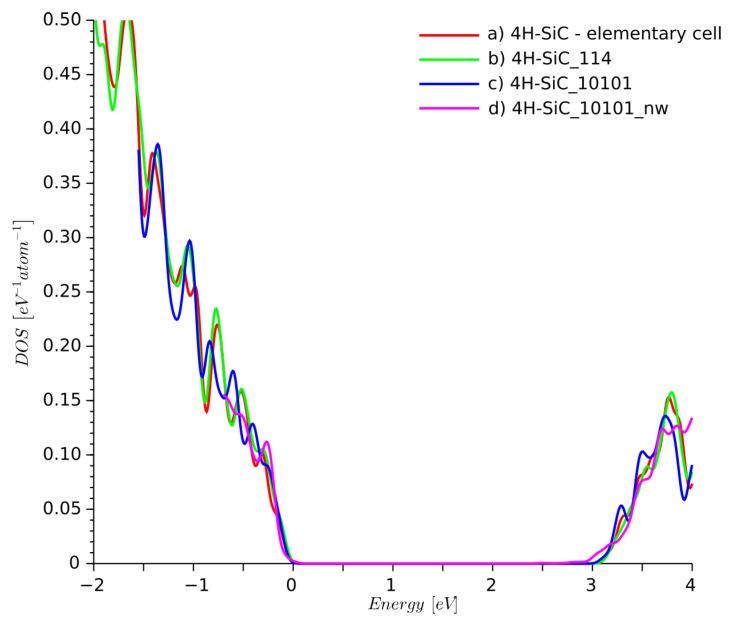
Density of states of ideal 4H-SiC for: (a) elementary cell, (b) 1×1×4 supercell, (c) 10×10×1 supercell, and (d) a 10×10×1 4H-SiC nanowire with side walls passivated by hydrogen atoms.

The DOS for 4H-SiC_10101_nw structure was summed up as a contribution of 20 atoms located in the center of the structure. The DOS plots obtained for all three supercells are similar in Figure 5. The DOS for supercells was calculated using the Gaussian smearing integration scheme, whereas the elementary cell DOS was obtained using the tetrahedron integration method.

## 4. Density of States for 4H-SiC with Defects

### 4.1. Point Defects

**Figure 6:**
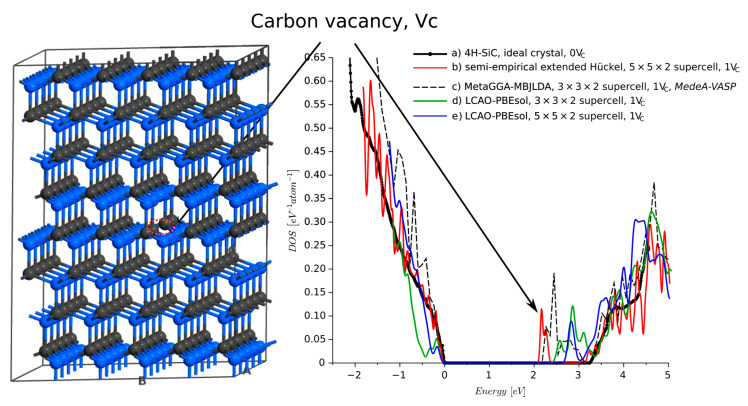
Density of states of 4H-SiC with carbon vacancy $V_{c}$.

The most important and stable point defect is the carbon vacancy defect, $V_{C}$ [4]. In previous studies [26,27], we used the DFT model with Meta-GGA MBJLDA functionals to show that $V_{C}$ introduces additional energy bands into the band-gap. Similar results were reported [28], where $V_{C}$ was a source of a band-gap narrowing and a new energy band. The authors considered a 4H-SiC 2×2×2 supercell with a carbon vacancy and found that $V_{C}$ introduced the energy band ca. 0.9 eV below the conduction band energy $E_{C}$. Here, we investigated a $V_{C}$ located in a 5×5×2 4H-SiC supercell (Figure 6). The 4.5 Å neighborhood of the $V_{C}$ was relaxed using the force fields with the Tearsoft1998 [29] set of potentials. To verify the results obtained from the semi-empirical calculations, in Figure 6 line (b), we quote results from [27], line (c). We also calculated the DOS using the DFT-LCAO approach with GGA-PBEs functionals and the DFT-1/2 correction [8] for different sizes of the supercell, lines (d,e). PW-HSE could not be used due to the large number of atoms considered. All these models predict the presence of a new energy band in the vicinity of $E_{C}$. The magnitude of DOS is also similar. According to our SEeH calculations, the energy band is located 0.9 eV below $E_{C}$, which is a similar value to that previously reported [28]. However, our previous calculations in MedeA-VASP and QuantimATK DFT-LCAO models predicted the energy band 0.8 and 0.2–0.5 eV below $E_{C}$, respectively. Another effect that can be observed is the narrowing of the band-gap. This is predicted by the semi-empirical calculations as well as the DFT-LCAO model. The carbon vacancy is often considered as explanation for Z1\Z2 traps [30], which are located ca. 0.7 eV below the $E_{C}$ [31,32]. As such, the depth of the energy level is overestimated by 0.2 eV by the SEeH. Similar (absolute) difference can be reported for the LCAO-PBEsol calculations. The energy level obtained from MetaGGA-MBJLDA Medea-VASP calculation is 0.8 eV below the minimum of conduction band. However, in this case, due to the computational limits the supercell is only 3×3×2 and calculations took more than 24 h (vs. 15 min for the extended Hückel approach).

### 4.2. Shockley Stacking Fault Defects

**Figure 7:**
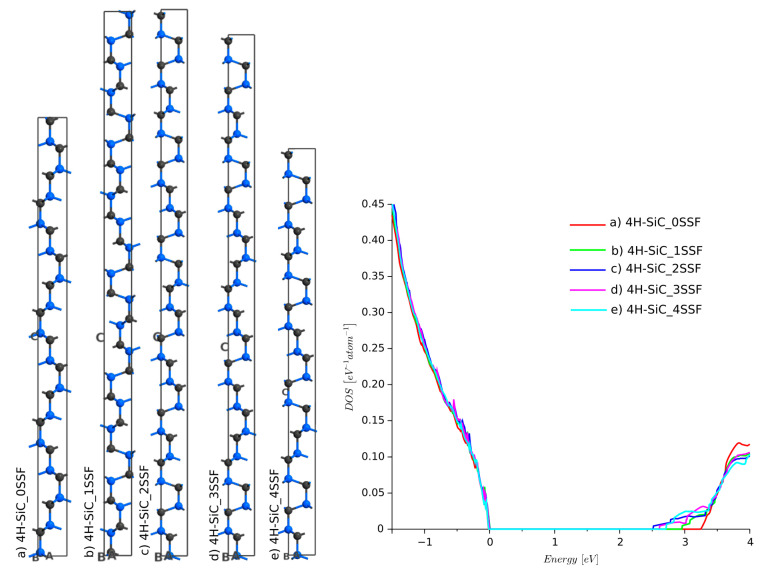
Density of states for 4H-SiC with Shockley stacking faults defects: (a) 4H-SiC_0SSF (reference structure with no defects), (b) 4H-SiC_1SSF, (c) 4H-SiC_2SSF, (d) 4H-SiC_3SSF, and (e) 4H-SiC_4SSF.

For the non-defected 4H-SiC, the stack of layers is ABCB [33]. An inappropriate sequence of Si–C layers causes stacking fault defects. There are many possibilities for these misalignments. The most crucial are Shockley stacking faults (SSFs) [5], shown in Table 2. The SSF defects lead to the degradation of SiC devices, which are sometimes called killing defects [34]. SSFs are dangerous since they may not be present during the fabrication process and can appear during a device’s normal operation. The change in the ABCB periodic sequence locally lowers the energy band-gap. A simulation employing semi-empirical extended Hückel was run for four different stacks, as shown in Figure 7. The DOS functions showed that SSF leads to a significant decrease in energy band-gap. The largest difference was found for the 4H-SiC_3SSF structure. The results reported here agree very well with our previous simulations [27], where MedeA VASP with the DFT framework and Meta-GGA with MBJLDA functionals were used.

### 4.3. Threading Edge Dislocations

Threading edge dislocation (TED) is a linear crystallographic defect with Burger vector $\frac{1}{3}\left[-2110\right]$ [35]. This type of dislocation is much harder to simulate since its presence disturbs the periodicity of the crystal. Thus, it is impossible to define only a small crystallographic cell and run simulations with periodic boundary conditions applied.

#### 4.3.1. Bulk Structure

**Figure 8:**
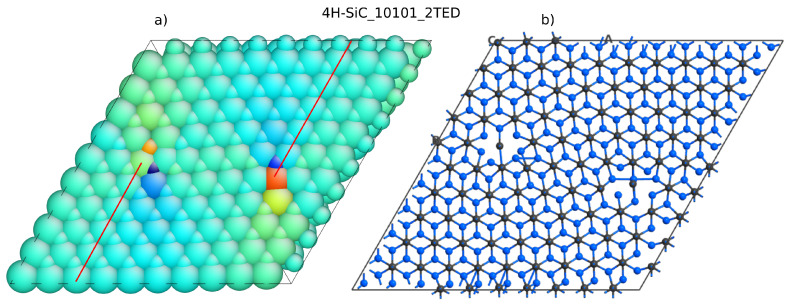
4H-SiC structure with two threading edge dislocations (TEDs): (**a**) strain colored atom shells and (**b**) atomic structure with bonds unveiled.

**Figure 9:**
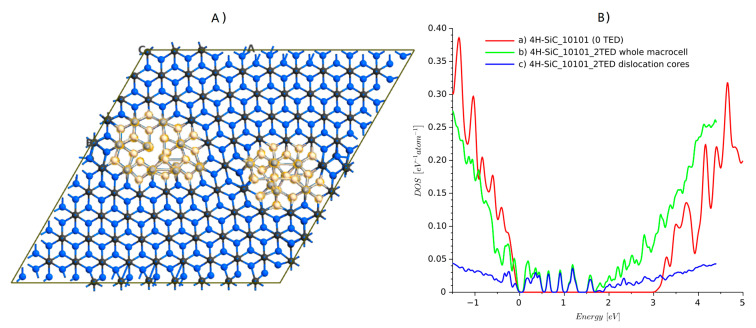
4H-SiC structure after relaxation and DOS plots: (**A**) relaxed atomic structure and selected atoms around dislocation core and (**B**) DOS plots.

Lazewski et al. [35] solved this problem by applying two TED defects with negative and positive Burgers vector. They created a supercell with a 8×6×1 elementary cell. The periodicity of this supercell was then recovered, and DFT simulations in VASP could be performed. There were almost 350 atoms included. This number of atoms is close to the computational limit of university-type servers. From our experience, we confirm that structures of more than 400 atoms are beyond the capabilities of popular server machines when DFT with GGA is applied. We repeated the calculation for a similar system containing two defects. The structure, shown in Figure 8, was created using Atomsk software [36]. It is a 10×10×1 supercell that contains 800 atoms in the case of the ideal structure. The structure with dislocation studied here has 810 atoms. Additional planes are marked by lines in Figure 8a and dislocation edge locations can be revealed by strain maps, which were visualized using Atomeye [37]. The dislocation introduced by Atomsk has to be relaxed first. Again, the force-fields calculation was used to relax the atom positions in the vicinity of the dislocation cores. The minimized structure, 4H-SiC_10101_2TED, is shown in Figure 9A).The DOS has also been calculated for the ideal crystal (structure 4H-SiC_10101).

Comparing both results showed that the structure with dislocations has a much lower energy gap, and dislocation edges introduce additional energy levels. This is clearly visible if only atoms around the dislocation edges are selected for DOS calculations. The dislocation edges are responsible for the modification of the DOS shape. The locations of additional energy levels are similar to those previously reported [35]. There is one maximum near the conduction band, another in the center, and the next 3–4 near the valence band. Notably, the computing time for the DOS calculations on eight parallel processors on a machine with 128 Gb RAM, 2× Intel Xeon CPU E5-2680 v3 (24 logical cores each) took ca. 4 h, which is less than one order of magnitude in comparison with the standard DFT approach.

#### 4.3.2. Nanowire Structure

**Figure 10:**
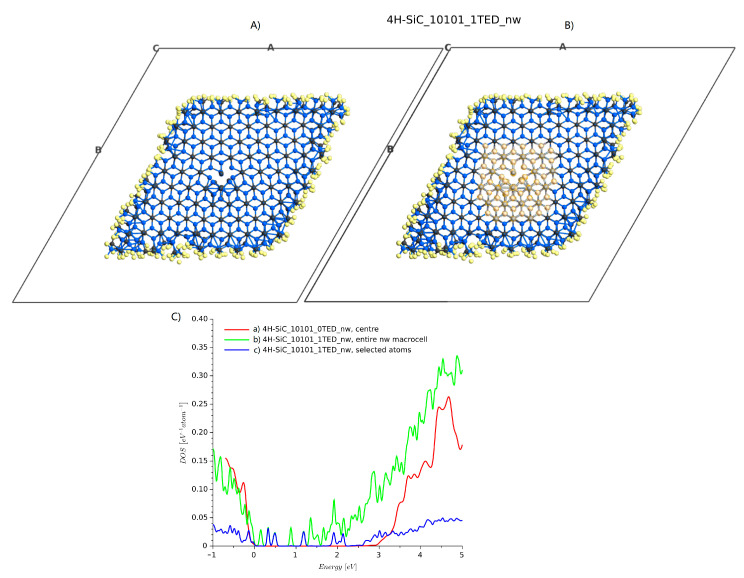
4H-SiC nanowire structure and DOS plots: (**A**) relaxed atomic structure, (**B**) selected atoms around the dislocation core, and (**C**) DOS plots.

The problem with supercells with a size of about 10×10×1 is that the defect cores interact with each other [35], and it is not possible to treat them individually. We tried to solve this problem by enlarging the cell dimensions by adding a space between side atoms and the edge of a cell. A geometry with a 10 Å margin is presented in Figure 10A. Considering the periodic boundary, infinite parallel nanowires were created. For this geometry, a single TED was introduced to the center of the domain. The external layers with hydrogen and the atoms near the dislocation core were relaxed. The total number of atoms was 970 (including hydrogen atoms used for passivation). The DOS summed from all atoms is shown in Figure 10C), line c. When all atoms are included, the energy band-gap is significantly reduced. This effect is a well-known property of SiC nanoparticles [38]. The additional DOSs are caused by nanowire edges. Calculating the DOS for the nanowire structure without any dislocation, Figure 4d and selecting atoms near the center of the stricture, a DOS similar to ideal bulk 4H-SiC is obtained (Figure 10B, line b). Then, considering the structure with a single dislocation, and again selecting the atoms near the dislocation edge, as shown in Figure 10B), the DOS (blue line, 4H-SiC_1TED_nw) is similar to that obtained for a bulk domain with two threading edge dislocations.

## 5. Summary and Discussion

Through this paper, step by step, we verified the accuracy and usefulness of the semi-empirical extended Hückel approach for efficient modeling of SiC atomic structures. None of the simulations required more than 4 h (with eight processors used in parallel). This computational time is more than an order lower than typical DFT GGA methods, but the DOS shape function is still similar. From the perspective of the solid-state engineering and semiconductor industry, it provides possibilities for fast and accurate investigation of non-ideal atomic structures. In this manuscript, we show that different polytypes of SiC, point defects, stacking faults, and threading edge dislocations can be treated by the semi-empirical extended Hückel approach. Future research will focus on other dislocation types: screw and basal plane dislocations. We would also like to verify the extended Hückel approach for GaN, the other wide band-gap semiconductor used in power electronics.

## Figures and Tables

**Table 1:** Energy band-gap $E_{g}$ calculated in QuatumATK using different models.

Model	Energy Band-Gap $E_{g}$ (eV)		
	3C-SiC	4H-SiC	6H-SiC
Extended Hückel semi-empirical with Cerda parameters	2.15	3.22	2.90
PW-HSE ^1^	2.27	3.23	2.95
LCAO-HSE ^2^	2.26	3.19	2.94
Reference values in the literature	2.36	3.23	3.00

^1^ PW-HSE: hybrid-functional calculations with plane-wave basis sets. ^2^ LCAO-HSE: hybrid-functional method for linear combination of atomic orbitals.

**Table 2:** List of Shockley stacking faults structures and calculated energy band-gaps. The stacking fault sequence is shown in bold.

Structure	Sequence of Layers	Number of Atoms	$E_{g}\left(\mathrm{eV}\right)$	$\Delta E_{g}\left(\mathrm{eV}\right)$
4H-SiC_0SSF	ABCB|ABCB|ABCB|ABCB	32	3.25	0.0
4H-SiC_1SSF	ABCB|ABCB|**CABA|C|**ABCB|ABCB	42	2.95	0.3
4H-SiC_2SSF	ABCB|ABC**A|BCAC|BC**|ABCB|ABCB	44	2.5	0.75
4H-SiC_3SSF	ABCB|A**CAB|CABA|C|**ABCB|ABCB	42	2.6	0.65
4H-SiC_4SSF	ABCB|A**CBC**|ABCB|ABCB	32	2.7	0.55

## Data Availability

The data presented in this study are available on request from the corresponding author.

## Abbreviations

The following abbreviations are used in this manuscript:

DFT	density functional theory
DOS	density of states
GGA	generalized gradient approximation
HSE	Heyd–Scuseria–Ernzerhof hybrid functional approach
LCAO	linear combination of atomic orbitals
LCAO-HSE	hybrid-functional method for linear combination of atomic orbitals
MBJLDA	modified Becke Johnson functional combined with local density approximation
MetaGGA	meta-generalized gradient approximation
PBEs	Perdew- Burke- Ernzerhof functionals for solids
PDOS	projected density of states
PW	plane wave
PW-HSE	hybrid-functional calculations with plane-wave basis sets
SD	screw dislocations
SEeH	semi-empirical extended Hückel
SSF	Shockley stacking fault
